# Interpolated functional manifold for functional near-infrared spectroscopy analysis at group level

**DOI:** 10.1117/1.NPh.7.4.045009

**Published:** 2020-11-27

**Authors:** Shender-María Ávila-Sansores, Gustavo Rodríguez-Gómez, Ilias Tachtsidis, Felipe Orihuela-Espina

**Affiliations:** a Instituto Nacional de Astrofísica, Óptica y Electrónica, Santa María Tonatzintla, Puebla, Mexico; b University College London, London, United Kingdom

**Keywords:** connectivity analysis, functional connectivity, functional near-infrared spectroscopy, manifold, topology

## Abstract

**Significance:** Solutions for group-level analysis of connectivity from fNIRS observations exist, but groupwise explorative analysis with classical solutions is often cumbersome. Manifold-based solutions excel at data exploration, but there are infinite surfaces crossing the observations cloud of points.

**Aim:** We aim to provide a systematic choice of surface for a manifold-based analysis of connectivity at group level with small surface interpolation error.

**Approach:** This research introduces interpolated functional manifold (IFM). IFM builds a manifold from reconstructed changes in concentrations of oxygenated $\Delta c{\mathrm{HbO}}_{2}$ and reduced ΔcHbR hemoglobin species by means of radial basis functions (RBF). We evaluate the root mean square error (RMSE) associated to four families of RBF. We validated our model against psychophysiological interactions (PPI) analysis using the Jaccard index (JI). We demonstrate the usability in an experimental dataset of surgical neuroergonomics.

**Results:** Lowest interpolation RMSE was 1.26e−4±1.32e−8 for $\Delta c{\mathrm{HbO}}_{2}$ [A.U.] and 4.30e−7±2.50e−13 [A.U.] for ΔcHbR. Agreement with classical group analysis was JI=0.89±0.01 for $\Delta c{\mathrm{HbO}}_{2}$. Agreement with PPI analysis was JI=0.83±0.07 for $\Delta c{\mathrm{HbO}}_{2}$ and JI=0.77±0.06 for ΔcHbR. IFM successfully decoded group differences [ANOVA: $\Delta {\mathrm{cHbO}}_{2}$: F(2,117)=3.07; p<0.05; ΔcHbR: F(2,117)=3.35; p<0.05].

**Conclusions:** IFM provides a pragmatic solution to the problem of choosing the manifold associated to a cloud of points, facilitating the use of manifold-based solutions for the group analysis of fNIRS datasets.

## 1 Introduction

Functional near-infrared spectroscopy (fNIRS) uses infrared light to probe indirect markers of brain hemodynamics.^1^ The continuous wave submodality continuously irradiates the scalp with near-infrared light often at several wavelengths. Attenuated backscattered light is detected with photodiodes. Light absorption changes are related to differential changes in oxyhemoglobin ($\Delta c{\mathrm{HbO}}_{2}$), deoxyhemoglobin (ΔcHbR), and total hemoglobin (ΔcHbT) that might be associated with neural activity. In some applications of fNIRS neuroimaging, the inference of brain activity at the group level is an important aspect of supporting or refuting the neuroscience hypothesis. Classical statistics has made an excellent work in allowing analysis of the cortical activity records.^2^ Random effects or second-level models are traditional avenues for group-level analysis excellent for affording quantitative answers related to confidence and goodness of fit over the regression models, the statistical significance of findings, etc. Although statistics suffices to answer most scientific demands, the classical approach has traditionally failed to provide easy data exploration critical for understanding cohort-level variability.

Manifolds are mathematical objects describing surfaces locally homeomorphic to some other normed space, which for practical purposes is almost always Euclidean. Manifold-based modeling approaches offer an alternative to observing full cohort variations both visually (upon rendering low dimensional embeddings) and quantitatively (based on the manifold geometry). Assuming that brain hemodynamics can be confined to a manifold, then the neuroimaging observations can be organized according to some criteria of distance, e.g., similarity, providing useful insights on brain activity as previously demonstrated.^3^^,^^4^ However, the analysis of the geometry and topology of a cloud of points faces important challenges, even though strong mathematical formalisms are already available.^5^ Consequently, this approach has remained largely underused and, as far as we are aware, mostly confined to the analysis of functional connectivity across neuroimaging modalities.^4^^,^^6^^,^^7^ Overall, the exploration of the variations of brain hemodynamics at the group level remains challenging.

This paper introduces interpolated functional manifold (IFM), a manifold-based analysis to generate a physiologically meaningful cartography of the group-level functional responses. The embedding of manifolds partially observed at point clouds to exploit only their visualization advantages often does not require the recovery of an explicit expression for the manifold. This facilitates usage but limits potential inference and/or intervention of the model to test different hypotheses. To make quantitative statements, the manifold must be navigated. Manifold navigation uses the geodesic, which can be estimated,^8^ but that can be calculated if an explicit analytical expression is available. In the context of fNIRS neuroimaging, we address here the problem of giving the cloud of points of observations over the functional manifold an explicit analytical expression to facilitate inference. The input to the proposed analysis is a set of fNIRS neuroimages. Following spatiotemporal processing to remove major artifacts, the brain hemodynamic responses are projected to a surface where the locations of the observations are organized by their similarity. A scalar field related to some hemodynamic function is then associated with the cloud of points and interpolated to yield an analytical expression. The output is a group-level descriptor of the hemodynamic function. This paper (i) introduces the formalization of IFM, (ii) establishes an empirical estimation of the error in building the surface, (iii) validates the new analysis by comparing its output to psychophysiological interactions (PPI) analysis, and (iv) exemplifies its use in an application domain from surgical neuroergonomics further providing nomological validity.

## 2 Methods

### 2.1 Interpolated Functional Manifold

#### 2.1.1 System input: the fNIRS neuroimages dataset

An fNIRS neuroimage fits a three-dimensional tensor in

Y(X,T,Π)={Δc(x,t,π)}, (1)

where X={1,…,X} indexes the discrete spatial locations at which the brain cortex is interrogated, i.e., the channels, T={1,…,T} indexes discrete temporal samples acquired, Π={π} indexes the physiological parameters, and Δc(x,t,π) represents a change in the concentration of parameter π at a location x and time t, respectively. This tensor can be augmented to a four-dimensional tensor Y(X,T,Π,N)={Δc(x,t,π,n)} with N={1,…,n} indexing a collection of neuroimages with N⊆S×P with P={1,…,p} representing the experimental units (participants, dyads, or other) and S={1,…,s} the (longitudinal or cross-sectional) recording sessions. For common bivariate fNIRS data $\Pi =\{\pi ,\pi ={\mathrm{HbO}}_{2},\mathrm{HbR}\}$, but some fNIRS data may also measure other chromophores, e.g., cytochrome-c-oxydase (CCO), that is $\Pi =\{\pi ,\pi ={\mathrm{HbO}}_{2},\mathrm{HbR},\mathrm{CCO}\}$.

#### 2.1.2 Projection to ambient space

Let Y^(X^,T^,Π^,N^) with X^⊆X, T^⊆T, Π^⊆Π, and N^⊆N, be a subtensor of Y(X,T,Π,N), denoted Y and Y^ for brevity. Subtensor Y^ unfolds as a tuplet like in Eq. (2):

Y^(X^,T^,Π^,N^)=⟨Δc(x^,t^,π^,n^)k⟩∀  x^∈X^,t^∈T^,π^∈Π^,n^∈N^, (2)

with k∈K=X^×T^×Π^×N^. Since the original rank of Y^ is 4, this unfolding to a vector shape alters the original lattice structure in Y^, but the tuple form is convenient as it permits using the known duality between vectors and points. The implications of such alteration are not further discussed here. Extracting a number j of subtensors Y^ from Y with j=1,…,J produces a collection of tuples that can be aggregated into a matrix sized J×K using

Y^J×K=[Y^1⋮Y^j⋮Y^J]=[Δc(x^,t^,π^,n^)1,1⋯Δc(x^,t^,π^,n^)1,k⋯Δc(x^,t^,π^,n^)1,K⋮⋱⋮⋱⋮Δc(x^,t^,π^,n^)j,1⋯Δc(x^,t^,π^,n^)j,k⋯Δc(x^,t^,π^,n^)j,K⋮⋱⋮⋱⋮Δc(x^,t^,π^,n^)J,1⋯Δc(x^,t^,π^,n^)J,k⋯Δc(x^,t^,π^,n^)J,K]. (3)

Any subtensor Y^ is susceptible for analysis, but often the interest shall be individual channels or region of interest (ROI) and perhaps temporal splitting by the blocks in the experimental stimulus train. For the rest of this paper, we use channel-based subtensors of the form Y^x,n(t^,π^) with π^={ΔcHbO2,ΔcHbR} and t^ chosen to match each of the task subperiods of the experimental blocks from onset to offset.

For two-dimensional subtensors, e.g., matrices, and exploiting the duality between vectors and points, orthonormal projection to an ambient space can be achieved by matrix multiplication of the pointset Y^ with the base coordinate system $E_{K\times K}=[e_{1},\ldots ,e_{K}]$ as per Eq. (4):

Yj×K=Y^j×kEk×k. (4)

Choosing $E=I_{K}$ with $I_{K}$, the identity matrix of range K yields an orthonormal projection to an ambient space where the Euclidean inner product induces a default (Euclidean) geometry $R^{K}$. We have shown that other projections are possible with implications both mathematically and for neuroscientific interpretation.^9^

**Fig. 1:**
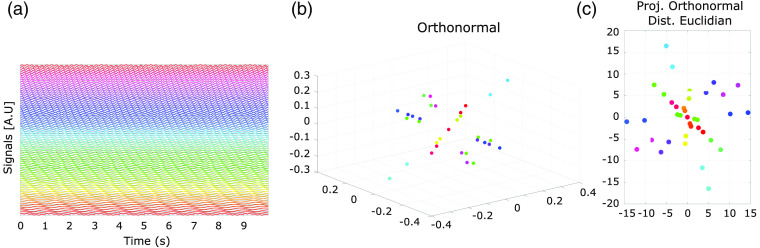
(a) A set of sinusoidals signals for exemplary purposes. (b) Exemplary orthonormal projection of sinusoidals signals. Each sinusoidal is represented by a point in the space. (c) Imposing an Euclidean geometry, distances among pairs of points (projected signals) can be estimated and used as surrogate as similiarity. Here, visual representation of all pairwise distances is achieved using classical multidimensional scaling.

Manifolds are spaces that are locally homeomorphic to some open unit disk. Arguably, the ones that have proved more common in data analysis are smooth (infinitely differentiable) manifolds which are locally Euclidean. If the manifold lives in some ambient metric space, the ambient distance function dictates the default geometry. We have previously studied the effect of different distance functions in supporting the construction of a manifold.^9^ Figure 1 shows an example of projecting fundamental sinusoidal to a euclidean geometry and the Euclidean distances between the sinusoidal.

When applied to a point set, the manifold-based analysis assumes the cloud of points $Y_{J\times K}$ to lie on a certain manifold M which has been observed only at $Y_{J\times K}$. An infinite number of surfaces may cross $Y_{J\times K}$ in $R^{K}$ and the choice of the one of convenience may be guided by different criteria, e.g., by topological stability^10^ or other.

#### 2.1.3 Manifold embedding

Manifold embedding assumes the cloud of points $Y_{J\times K}$ to lie on a manifold. The classical singular value decomposition (SVD) of a real data matrix $Y_{J\times K}$ provides one of such embeddings and is given by $Y=U\Sigma V^{T}$, where $U=[u_{1},\ldots ,u_{j}]\in R^{J\times J}$ and $V=[v_{1},\ldots ,v_{k}]\in R^{K\times K}$ are orthogonal matrices and $\Sigma =\mathrm{diag}(\sigma_{i})\in R^{j\times K}$ where $\sigma_{1}\geq \sigma_{2}\geq \cdots \geq \sigma_{l}\geq \cdots \geq \sigma_{L}\geq 0$, L=min{J,K}. Then, choosing M<L, a matrix of lower rank than Y is obtained as

$${\overset{˜}{Y}}_{J\times M}=\overset{M}{\underset{m=1}{\sum }}u_{m}\sigma_{m}v_{m}^{T}.$$ (5)

${\overset{˜}{Y}}_{J\times M}$ is a projection of $Y_{J\times K}\in R^{J\times K}$ onto a space $R^{J\times M}$. Different embeddings result in distinctly different ${\overset{˜}{Y}}_{J\times M}$ and distortions of M. We have used Isomap in the past.^4^ Here, we opted for SVD for simplicity, and because strengths and weaknesses are well understood. Reviews on dimensionality reduction are available,^11^ and deeper discussion is beyond the scope of this work.

#### 2.1.4 Setting a scalar function

Thus far, fNIRS reconstructed images have been transformed into a cloud of points in an ambient space and then embedded into a concise subspace. The manifold M is only known at j=1,…,J observations $Y_{J\times K}$ and its embedding ${\overset{˜}{Y}}_{J\times K}$. However, note that Eq. (5) only yields the new loci of $Y_{J\times K}$, but it does not produce an analytical expression of the manifold surface. We associate a scalar function to the cloud of points $Y_{J\times K}$ such that the function has some physiological interest.

Let $Z=\{(Y_{j,k},z_{j})\}$ where $Y_{J\times K}$ is the set of J points that have been observed and $z_{j}\in R$ some scalar quantity. This surface is projected alongside the manifold to the ambient space $R^{M}$, $\overset{˜}{Z}=\{({\overset{˜}{Y}}_{j,m},z_{j})\}$. Then, Z is a scalar function discretely defined at the observations in M. In this case, ${\overset{˜}{Y}}_{j,m}$ represents the location of one observation in the manifold and $z_{j}$ represents a chosen descriptor related to brain hemodynamics. Here, we chose $z_{j}$ to be the area under the curve of one ΔcHb species. Suitability of variations of the approach using other descriptors shall depend on the research question at hand.

#### 2.1.5 Radial basis functions interpolation

The final step to retrieve an explicit model for the scalar surface is an interpolation, here in terms of RBF. For our purposes, RBF have two advantages over classical interpolation: they are algorithmically simpler to escalate with larger datasets and they are better suited to cope with the curse of dimensionality.

Let $Y_{i,K}\in R^{K}$ be a nonobserved location of the (assumed) hemodynamic surface. We want to find a continuous function s: $R^{K}\to R$, which satisfies the interpolation conditions; for all $Y_{j,k}$, $z_{j}$, the function ought to be valued $z_{j}=Z(Y_{j,k})$. The radial approximation at $s(Y_{i,k})$ is given by $S(Y_{i,K})=\overset{J}{\underset{j=1}{\sum }}\beta_{j}\Phi ({\left\|Y_{i,K}-Y_{j,K}\right\|}_{2})+p(Y_{i,k})$ where $\beta_{j}$ are the unknown model coefficients to be determined. Here, $\Phi ({\left\|\cdot \right\|}_{2})$ is any RBF, ${\left\|\cdot \right\|}_{2}$ is the Euclidean norm, and $p(Y_{i,k})$ is a regularizing polynomial. The degree of the polynomial $p(Y_{i,k})$ depends of the RBF selected. For a semi-positive RBF, $p(Y_{i,k})$ guarantees the nonsingularity of the interpolation matrix.^12^ When the RBF is positive definite, the interpolant does not require a regularizing polynomial, i.e., $p(Y_{i,k})=0$. The following positive definite RBF Φ(r,c) in Eq. (6) have been tested here:

$$\text{Gaussian}\;(\mathrm{GA}):\;\Phi (r,c)=\exp[-{\left(cr\right)}^{2}],$$ (6a)

$$\text{Multiquadric}\;(\mathrm{MQ}):\;\Phi (r,c)=\sqrt{1+{\left(cr\right)}^{2}},$$ (6b)

$$\text{Inverse Multiquadric}\;(\mathrm{IMQ}):\;\Phi (r,c)=1/\sqrt{1+{\left(cr\right)}^{2}},$$ (6c)

$$\text{Marten}\;(\mathrm{MR}):\;\Phi (r,c)=\exp[-(cr)][3+3cr+{\left(cr\right)}^{2}].$$ (6d)

In these, $r={\left\|Y_{i,K}-Y_{j,K}\right\|}_{2}$ and c is a shape parameter that may be optimized by means of Rippa algorithm.^12^ A J×J linear system of equations in $\beta_{j}$ is obtained whose interpolation matrix is given as

$$s=[\Phi ({\left\|Y_{i,K}-Y_{j,K}\right\|}_{2})].$$ (7)

IM is a symmetric matrix. The solution to the interpolation problem gives an analytic expression of the hemodynamic surface. Note that interpolation here is made in the high dimensional manifold and not on its embedded counterpart. Rejection of channels by any data quality control strategy may alter the cloud of points from which the surface is recovered, yet the model itself remains the same.

Meshfree methods, particularly those based on RBFs, are often better adapted to deal with changes in the geometry of domain of interest. Meshfree discretization techniques are based only a set of independent points and can handle a huge number of dimensions in comparison to traditional methods that are mostly limited to three-dimensional problems. To find the RBF interpolator of the set of data requires the solution of a system of linear equations with a dense n×n matrix, these matrices tend to be rather ill-conditioned. In some cases, to address this problem, it is necessary to use alternative interpolation RBF strategies. In our case, to maintain the conditional matrix well-conditioned, we tuned the shape parameter for RBF approximation using Rippa strategy.^12^

### 2.2 Experimental Setup

**Fig. 2:**
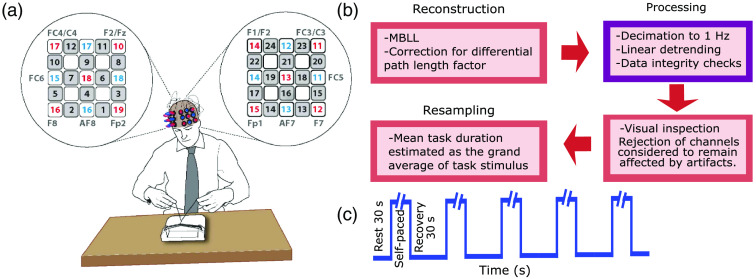
(a) The observed region and the location of sources (red), detectors (blue), and channels (black with gray background) with targeted scalp positions. (b) Flowchart summarizing the signal processing. (c) Stimulus train. The task was self-paced and hence the timing varied. Subfigure (a) is previously unpublished but it is the property of the original authors of the Imperial group study who kindly gave us permission to use it.

The fNIRS experimental dataset used for demonstration purposes has been described elsewhere.^4^ Briefly, the dataset was aimed at investigating surgical skill acquisition in a cohort of surgeons with varying degrees of expertise. Ethical approval for the original collection of this dataset was granted to the research group that originally collected the data at Imperial College London by the local Research Ethics Committee. The research group at Imperial College London obtained written informed consent from each participant before enrollment during the data collection period.^4^ Sixty-two surgeons participated (19 consultants, 21 trainees, and 22 medical students). The task consists of performing four throws of hand-tied surgical reef knots. The experiment followed a block design with a period of baseline motor rest (30 s) followed by five blocks of the task (self-paced surgical reef knot) and recovery periods (30 s). fNIRS neuroimages were acquired at 10 Hz using a 24-channel Optical Topography System (ETG 4000, Hitachi Medical Co., Japan) at 690 and 830 nm. Optodes were placed bilaterally over the prefrontal cortices, positioned according to the International 10-20 system maintaining 3 cm of interoptode distance. The observed region covers the prefrontal cortex. Positioning of channels targeted the area enclosed by Fp1 (fixed point), F7, FC3/C3, and F1 in the left side, and analogous area enclosed by Fp2 (fixed point), F8, FC4/C4, and F2 in the right side. The area covered includes the dorsolateral prefrontal cortex where according to the original work activity was expected. This area is related to movement planning and decision making, high order function evoked by the knot-tying stimulus. Figure 2 shows the region covered and the signal processing flowchart. The reader is referred to the original Ref. 4 for further details.

### 2.3 Data Processing

Neuroimages were reconstructed using the modified Beer–Lambert law with correction for differential path length factor. Processing consisted of signal decimation to 1 Hz to reduce systemic influence, linear detrending to remove system drift and data integrity checks were carried out in ICNNA v1.1.3.^13^ Integrity checks included Haar wavelet-based detection of motion-related artifacts, as well as saturation detection individually at both wavelengths as already described.^14^ These automated integrity checks were complemented with visual inspection by one of the researchers and subserved rejection of channels considered to remain affected by artifacts. The dataset was not accompanied by short channel readings to further remove scalp blood flow artifacts.

Since this is a self-paced task, each subtensor was resampled over T to the overall mean task duration throughout the dataset. Mean task duration $\overset{¯}{t_{\text{task}}}$ was estimated as the grand average of task stimulus subperiods across all subjects and blocks. The length of the unfolded resampled tuples was $K=2\cdot \overset{¯}{t_{\text{task}}}(\overset{¯}{t_{\text{task}}}$ samples × 2 Hb species), the first half corresponding to $\Delta c{\mathrm{HbO}}_{2}$ and the second half to ΔcHbR.

The new IFM analysis was performed with bespoken scripts in Matlab (R2016a, Mathworks) and Mathematica (11, Wolfram Research). Statistical analysis was carried out also in Matlab.

### 2.4 Estimation of Error and Assessment of the RBF

The explicit manifold expression for the experimental dataset is not available, and hence, the performance of the approach is evaluated by subsampling the available observations assuming that the expression retrieved from using the full dataset was the closest to the real solution. Subsamples of the dataset were picked randomly by splitting the dataset into two subsets, one for model parameter learning (train set) and the other for the assessment (test set). Following a bootstrapping strategy, four scenarios were prepared to split the available dataset into train-test partitions: 70% to 30%, 60% to 40%, 40% to 60%, and 20% to 80% of train and test sets, respectively. The number of replications were 10 and the root mean squared error [Eq. (8a)] was used as a measure of global error, and the local absolute errors [Eq. (8b)] was used for map differences:

$$\mathrm{RMSE}=\sqrt{\frac{\overset{J}{\underset{j=1}{\sum }}{\left[s(Y_{j,K})-Y_{j,K}\right]}^{2}}{J}},$$ (8a)

$${\text{Absolute Error}}_{j\in J}=|s(Y_{j,K})-Y_{j,K}|.$$ (8b)

### 2.5 Validation

Connectivity data originate from the experimental dataset described in Sec. 2.2 Although IFM is not bounded to segregational activity or integrational, e.g., connectivity, analysis, here we emphasize its integrational capacity. Hence, we compared ourselves to a functional connectivity analysis approach. Concurrent validity was established by comparison of result similarity against PPI.^15^ PPI is a variant of the general linear model that includes a specific regressor expressing the interaction between a psychological variable (task design) and physiological variable (the time series of brain region). It is a functional connectivity analysis method based on classical statistics. Although the original definition of the PPI model is seed-based, it is trivial to explore brain connectivity by repeating the analysis over channels in a pairwise manner. Also, the psychological variable in PPI implies that it was meant to be defined over active channels.

Since the output of PPI is a graph, to enable comparison, we enforce the isolation of one graph from the manifold. Manifolds are more expressive than graphs, e.g., a manifold might contain infinite graphs, and hence different criteria can be used to pick one graph. For the comparison with PPI, we look for the greatest match between connectivity network of PPI and connectivity networks of IFM (estimated at different neighborhood sizes), using the Jaccard index as a measure of similarity. The neighborhood size ε was discretely allowed to vary in small steps from the smallest (size 0, i.e., all points are isolated components) to the biggest diameter of the point cloud (one single component). A connectivity graph was retrieved for every group and every value of ε. The ε maximizing (on average) the agreement between the two approaches provides a good guide for establishing concurrent validity during comparison with PPI.

Finally, for comparison between the graphs, the Jaccard index^16^ was used to establish the similarity between the graph solutions found by PPI and IFM. The Jaccard index is the ratio of the intersection over the union of two sets, and when applied to graphs it is computed over the sets of edges.^17^

Given the nonstatistical nature of IFM, we further suggest an alternative graph isolating algorithm that can be used in circumstances when no other standard is present, yet a graph is required as a solution. This alternative algorithm looks for a topologically stable region by examining the change in the number of connected components in the graph and choosing the one that lives longer. For each neighbor size, the number of connected components of the point cloud is calculated. Then, intervals of neighborhood sizes are generated where the number of components is stable, and the largest (widest) interval is assumed to represent the most stable topology. The midpoint of this interval provides the more topologically stable neighborhood size. This approach is based on the number of connected components. Although it may be considered related to topological data analysis,^5^ it does not account for the persistence of higher-order topological features (holes and voids).

## 3 Results

### 3.1 Analysis of Segregated Activity

IFM, as presented here, is not confined to answer connectivity questions alone. Activity analysis can be achieved for instance by projecting to the manifold a synthetic point encoding the convolution of a given stimulus train with some hypothesized hemodynamic response function. However, since PPI is an evoked activity-based connectivity analysis, we shall constraint graph isolation later to active channels. Thus, for reference, a classical task minus baseline analysis of segregated activity was conducted^13^ and the channels found as active here were later used to crop the graphs during validation. Segregational activity was established following a task minus baseline analysis. The reconstructed neuroimages were split into segments corresponding to blocks including the baseline of 20 s, and the task periods. Timecourses of the baseline and task periods across all blocks were then rearranged into two random variables; baseline and task. A paired two-sample Wilcoxon Sign Rank test, with significance level of 0.05, was used to establish significance independently for each Hb species. The aggregated combination of the test for the ${\mathrm{HbO}}_{2}$ and the test for the HbR produced a single pattern that can be related to activity (coupled significant increase in ${\mathrm{HbO}}_{2}$ and significant decrease in HbR).

**Fig. 3:**
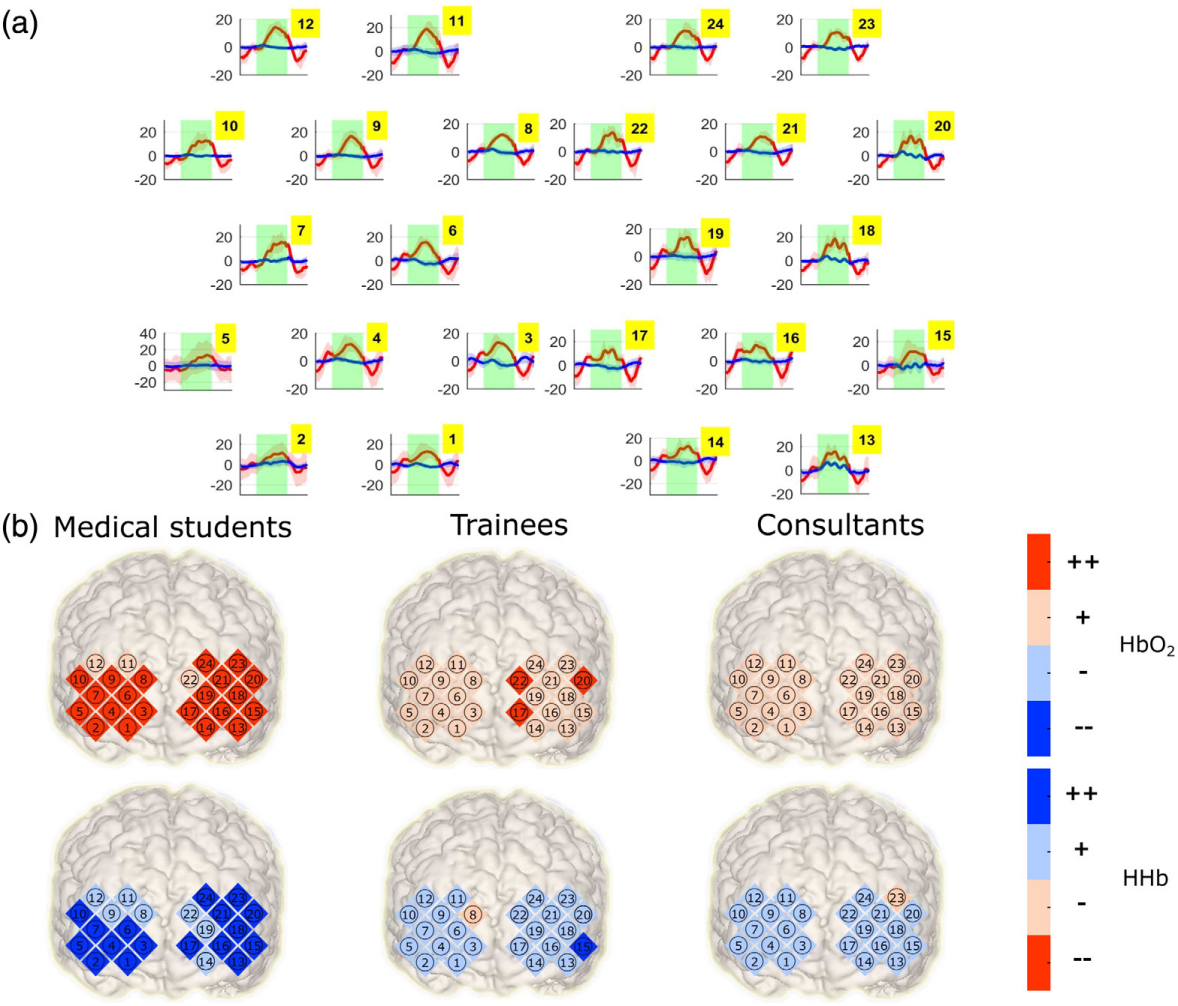
Exemplary fNIRS neuroimage of the dataset. (a) Block averaged time courses of the $\Delta c{\mathrm{HbO}}_{2}$ (red) and ΔcHbR (blue) block averaging is used here for illustration purposes only, but data analysis proceeded without block averaging. Standard deviations across blocks are represented by shaded reddish and blueish regions, respectively. Green patches indicate the task subperiod. The approximate geometric distribution of the channels during acquisition is respected here. The characteristic increase in $\Delta c{\mathrm{HbO}}_{2}$ coupled with a decrease in ΔcHbR associated with functional activation can be appreciated in several channels. Units are [mol]. (b) The activity matrix from a task minus baseline segregational analysis projected on a brain. The projection is approximate according to the locations of the recorded channels (no registration efforts have been made in producing this plot). Brain activity is established by a statistical comparison of the observations during the task and baseline periods. (++) statistically significant increment; (+) nonsignificant increment, (−) nonsignificant decrement, and (–) significant decrement where the significance threshold here has been chosen at p<0.05.

An example of a block-averaged neuroimage from the dataset is shown in Fig. 3(a). Figure 3(b) shows the active channels during the performance of surgical reef knots in a brain as established by the task minus baseline paradigm. Each brain represents the average activity of a surgeon group according to their experience. Analysis of activity is carried out separately for each hemoglobin species.

### 3.2 Variance Maintained during Embedding

**Fig. 4:**
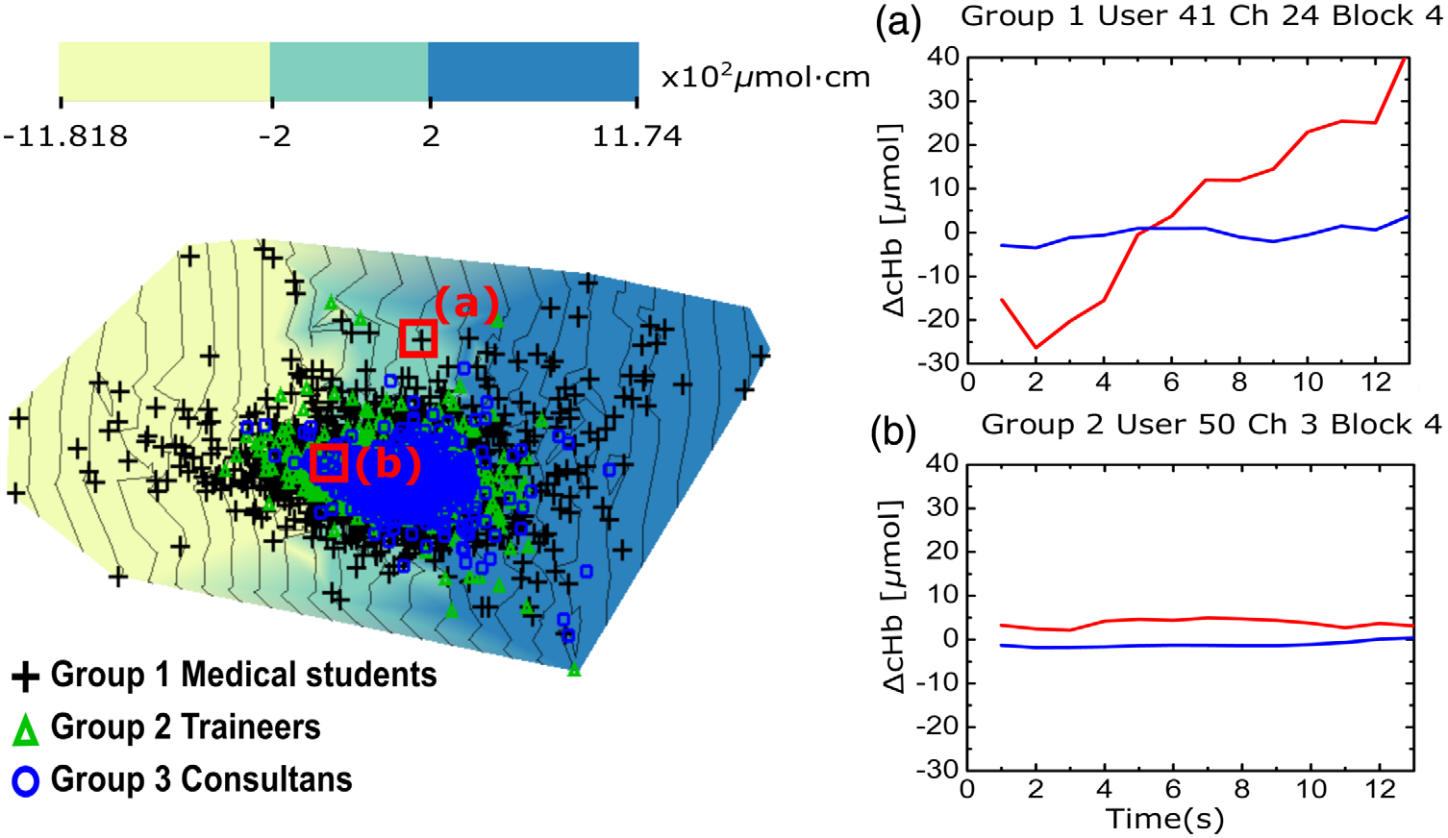
(Left) The hemodynamic manifold of a cohort of surgeons while performing a knot tying task. Points in the manifold correspond to 26-dimensional channel-based subtensors of the fNIRS neuroimaging dataset; 13 samples corresponding to $\Delta c{\mathrm{HbO}}_{2}$ and the other 13 to the ΔcHbR. Point markers have been colored and marked according to the original participant surgical skill. The distribution of the points associated with the subtensors in the ambient space is self-organized according to the pairwise distances. Closer points indicate more similar signal behavior. The color encodes the intensity of the scalar field associated with the area under the curve of the $\Delta c{\mathrm{HbO}}_{2}$ subvector. (Right) Two randomly picked exemplary points (a) and (b) help to interpret the embedding. The underpinning original subtensors associated to these points are shown on the right: $\Delta c{\mathrm{HbO}}_{2}$ (red) and ΔcHbR (blue).

Cardinalities for the dataset in Sec. 2.2 are; subjects #P=62, sessions #S=1 (cross-sectional), fNIRS neuroimages #N=#P·#S=62, #X=24 channels per image, and hemodynamic parameters #Π=2, i.e., $\Delta c{\mathrm{HbO}}_{2}$, ΔcHbR. The task was self-paced, with the longest recording being #T=474 samples long. The mean task duration was found to be $\overset{¯}{t_{\text{task}}}=13$ samples per hemoglobin species. As described in Sec. 2.3 we choose our subtensors based on channels, and thus, for this exercise, we project orthonormal to an ambient space K=26 (13 samples × 2 Hb species) with one point per channel from each neuroimage. This 26-dimensional manifold cannot be directly visualized. The manifold was embedded into a K^=2 dimensional space convenient for visualization according to Sec. 2.1.3. Figure 4 shows the manifold embedded in this two dimensional space. Variance maintained during the dimensionality reduction was 39.70±0.52%. Different choices of subtensors under the orthonormal projection shall project to different ambient spaces, and thus the maintained variance must be reported whenever using IFM. Embedding is an ill-posed problem with infinite solutions; rotations, flipping, and translations of a given configuration also constitute valid solutions to the embedding, but it is notable that under a given embedding strategy, the variance maintained is not affected. Note that the manifold does not represent the topographical distribution of the channels as located on the head or over the brain, but it is expressed in a space of signal similarity.

In terms of neuroscience, there are two major semantics in the manifold: first, the global semantics is governed by the major response to a stimulus. Responses of brain regions that manifest any increase or decrease of the Hb species will tend to move toward the periphery of the manifold, whereas flatten responses are gathered around a conceptual center (see Fig. 4 subplots). Since the manifold may be flipped or rotated every time, the specific region of the manifold where segregated activity can be found may change, but it will be confined to some quarter of the observed manifold. Some exploration of the manifold is needed to locate this region, but with some practice, this becomes straightforward. In the specific case of Fig. 4, this region was the north-west of the projection. Channels on this region of the manifold, i.e., active channels, were mostly those in the dorsolateral PFC and more pronounced in novices in clear nomological agreement with literature^18^ and perfectly matching with the original study.^4^ Second, the local semantics is governed by similarity in the responses. The local regions with co-active behavior are expected to exhibit hemodynamic timecourse patterns that are more similar among themselves that against noncoactive regions. Coactive regions attract themselves during the building of the manifold, whereas noncoactive regions repel themselves. The result is that the neighborhood of some channel response includes the response of those other channels exhibiting the same evoked hemodynamics. Exploring the manifold revealed a lateralized response (not shown) with slightly higher left activity. This coincides with the supporting segregational activity analysis by classic task minus baseline analysis in Fig. 3 as well as with the mixed effects model used for validation below. This may only be the consequence of the predominance of right-handed participants.

### 3.3 Error of RBF Interpolation

**Fig. 5:**
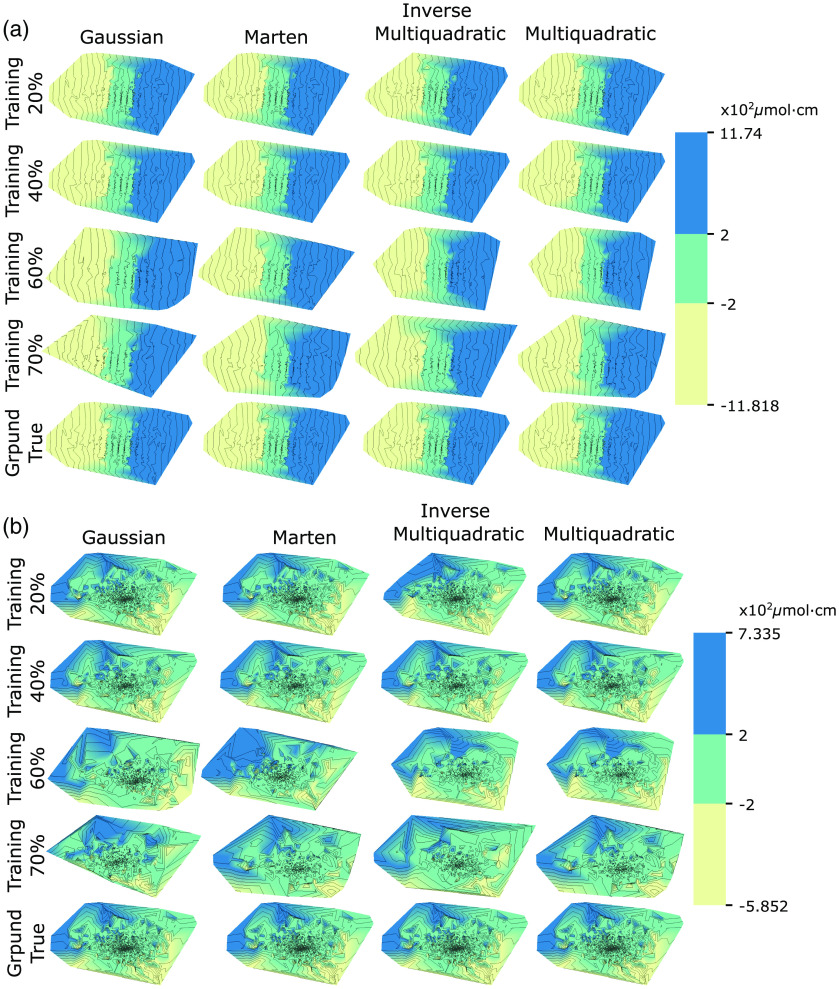
Parametric surfaces reconstructed with the different RBF. (a) $\Delta c{\mathrm{HbO}}_{2}$ and (b) ΔcHbR. Each column corresponds to a different RBF. For each subsampling scenario, rows correspond to approximated interpolations. Being piecewise interpolation, training with fewer samples yields smoother surfaces without severely penalizing the error. The orientation of the surfaces is arbitrary as dictated by the SVD projection, and results from the ill-posed problem of dimensionality reduction in which flipped or rotated solutions are equivalent. A trend is apparent in most cases; along with the main component for $\Delta c{\mathrm{HbO}}_{2}$ and tilted over the two main components for ΔcHbR.

Table 1 summarizes the estimated errors associated to the different interpolations. Multiquadratic RBF has the smallest associated error. However, no statistical differences were found among RBF families neither for the $\Delta c{\mathrm{HbO}}_{2}$ [ANOVA: F(3,12)=1.4804, p=0.269] nor the ΔcHbR [ANOVA: F(3,12)=1.005, p=0.423] surfaces. Given that the differences are negligible among different RBFs, Marten RBF interpolation appears more suitable for interpretation in the sense of producing a less bumpy surface with only a minor increment in error. Figure 5 shows the surfaces approximated using each RBF under every scenario for error estimation for $\Delta c{\mathrm{HbO}}_{2}$ and ΔcHbR, respectively. The parametric map for $\Delta c{\mathrm{HbO}}_{2}$ shows an apparent east-west gradient (along with the first component of the embedding). For ΔcHbR, the trend is also apparent, but the gradient is attenuated, and the direction appears tilted over the components of the embedding. Despite ill-posedness, the trend itself being related to variance is meaningful and exist regardless of the solution. We did not normalize for the expected numerical dominance of the oxygenated haemoglobin for which concentration changes will be about three times those of the reduced haemoglobin. Hence, the alignment of the $\Delta c{\mathrm{HbO}}_{2}$ gradient along the first component is expectable.

**Table 1:** Root mean squared error. Mean (μ) ± standard deviations (σ) across replications are indicated.

Train	Test	Multiquadratic	Inv. multiquadratic	Gaussian	Marten
${\mathrm{HbO}}_{2}$					
70%	30%	3.24e−4±6.35e−8	6.16e−4±2.96e−7	7.12e−02±3.31e−02	2.27e−4±3.30e−8
60%	40%	1.22e−4±2.21e−8	8.84e−5±7.41e−9	6.37e−03±1.02e−4	6.34e−5±2.83e−9
40%	60%	9.14e−5±6.26e−9	6.75e−4±1.01e−6	3.58e−03±1.14e−5	1.38e−4±1.27e−8
20%	80%	3.62e−5±1.03e−9	1.01e−4±2.43e−8	1.73e−03±7.76e−7	7.70e−5±4.14e−9
Average		1.43e−4±2.32e−8	3.70e−4±3.52e−7	2.70e−02±8.30e−03	1.26e−4±1.32e−8
HbR					
70%	30%	6.31e−7±1.82e−13	2.16e−6±5.50e−12	4.01e−02±1.76e−9	7.66e−7±2.20e−13
60%	40%	4.18e−7±3.60e−13	4.01e−6±1.19e−10	5.68e−5±9.18e−9	3.09e−7±8.44e−14
40%	60%	5.64e−7±4.75e−13	5.91e−7±3.16e−13	2.32e−5±1.17e−9	5.30e−7±2.95e−13
20%	80%	1.05e−7±1.04e−14	6.97e−7±7.13e−13	4.59e−6±4.40e−12	2.59e−7±4.90e−14
Average		4.30e−7±2.50e−13	1.87e−6±3.15e−11	3.12e−5±3.03e−9	4.66e−7±1.62e−13

**Fig. 6:**
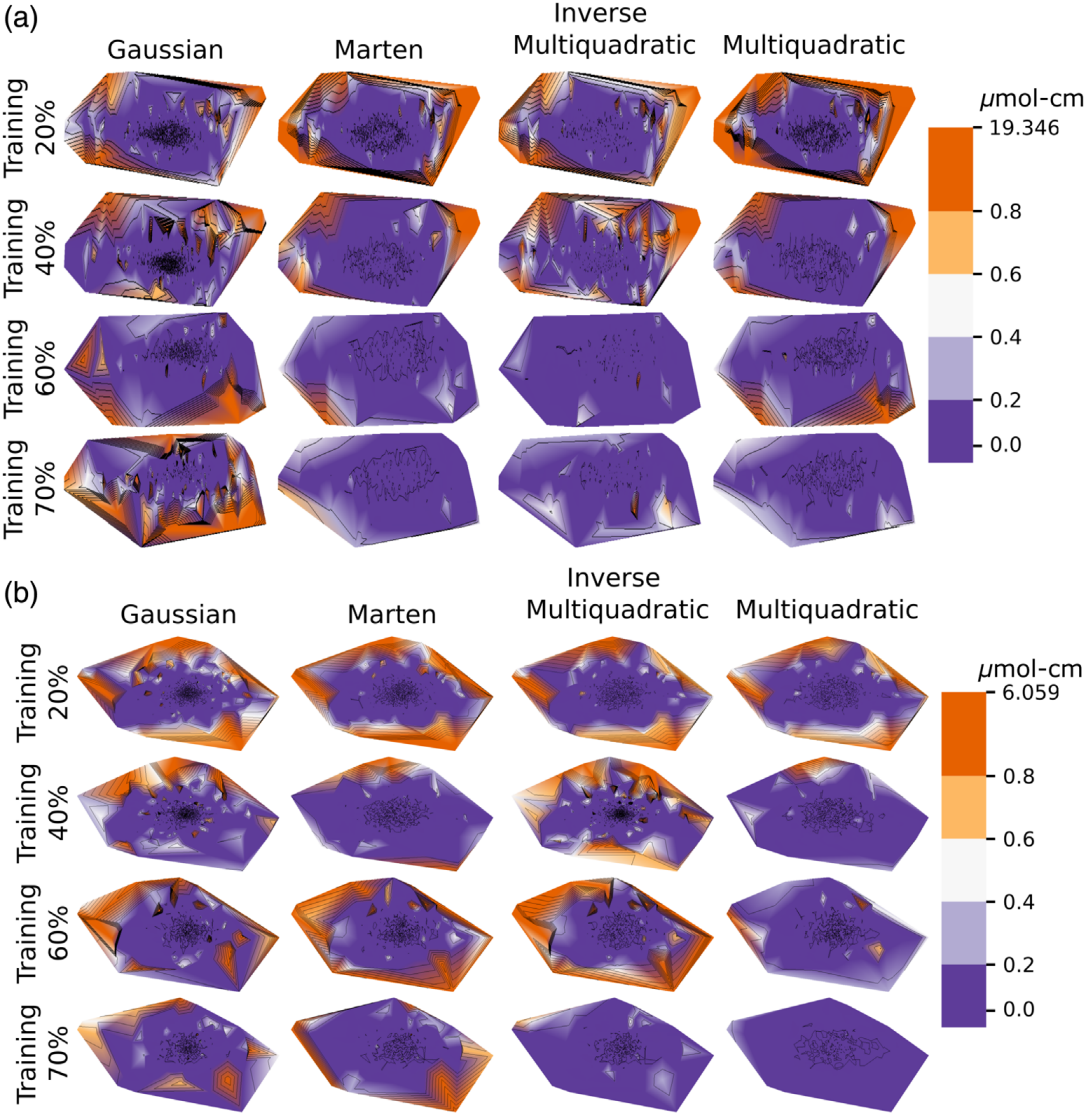
Error maps illustrating the difference of the surfaces recovered with subsampling and the full surface. (a) Refers to the difference of surfaces for ${\mathrm{HbO}}_{2}$ and (b) To the difference of surfaces for HbR. The absolute error is shown. Error is smaller in the central region of the manifold since this region is densely populated

The maps of errors are shown in Fig. 6. These illustrate the differences between the surfaces recovered with subsampling and the surface of the full dataset. The periphery of the manifold where the density of the observations is sparser is naturally exposed to higher errors.

### 3.4 Validation

Concurrent validity was established by computing the agreement between the approaches according to the Jaccard index. We have compared ourselves against two other approaches. First, against a classical mixed effect model, and second, against, PPI.

#### 3.4.1 Validation against a mixed effect model

**Fig. 7:**
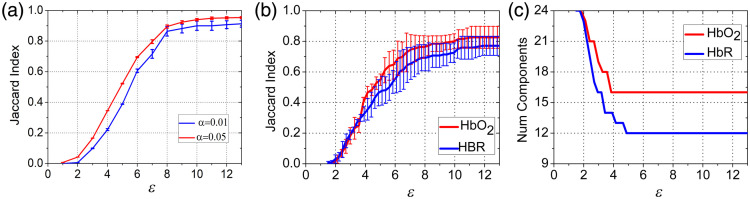
Validation. (a) Agreement with mixed effects model for $\Delta {\mathrm{HbO}}_{2}$. Variations in matching with the model output for different neighborhood sizes. (b) Agreement with PPI. Variations in matching with the PPI output for different neighborhood sizes. (c) Values of the midpoint of the most stable interval with respect to the number of components of the graphs.

A mixed effect model was build using SPM-NIRS software. In this model, the random effect regressors encode group variances. Contrasts were defined over the fixed terms to generate the groupwise activity maps using the F statistics at α=0.05 and α=0.01 significance level. Full connectivity was assumed among statistically significant active channels. IFM was compared against the resulting graphs. The Jaccard index is calculated as the sum of the edges in the active channels sets $E_{\mathrm{PPI}}$ and $E_{\mathrm{IFM}}$ of the upper triangular part of the adjacency matrices: $\mathrm{JI}=\#(E_{\mathrm{PPI}}\cap E_{\mathrm{IFM}})/\#(E_{\mathrm{PPI}}\cup E_{\mathrm{IFM}})$. The results are summarized in Fig. 7. JI reached 0.89±0.01 and 0.86±0.01, respectively.

#### 3.4.2 Validation against PPI

Agreement between the two graph isolation approaches and PPI as measured with the Jaccard index over 50 repetitions is reported in Table 2. Both graphs isolating approaches are reported in this table, the sequential seek ε maximizing matching and the one inspired in topological stability. The progression in the agreement as a function of the neighborhood size as well as the number of connected components in the graph is shown in Fig. 7.

**Table 2:** Summaries of similarities with PPI under both graph isolating algorithms Jaccard index and associated neighborhood sizes ([min. max.]) are reported.

	Sequential seek		Topological stability	
	Jaccard index (μ±σ)	ε	Jaccard index (μ±σ)	ε
${\mathrm{HbO}}_{2}$	0.83±0.07	11.12	0.79±0.04	9.81 [3.85-15.77]
HbR	0.77±0.06	11.89	0.70±0.07	9.11 [4.88-13.33]

**Fig. 8:**
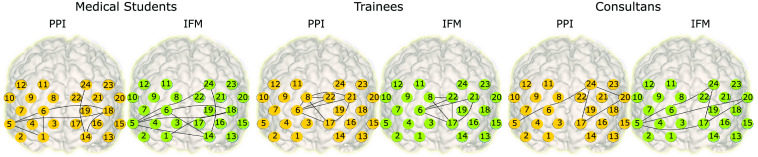
Expertise related group networks and validation of IFM against PPI on experimental data for $\Delta {\mathrm{HbO}}_{2}$. The cortical networks for both techniques for the different expertise groups are shown. IFM provides a richer representation (e.g., the manifold contains infinite graphs), but for visual comparison, the networks shown for IFM were thresholded to maximize the Jaccard index pairing to the PPI.

Despite the different nature of the approaches -one statistical, one topological-, agreement with PPI analysis can be as good as 0.83±0.07 for $\Delta cs_{2}$ and 0.77±0.06 ΔcHbR in terms of Jaccard supporting our claim of concurrent validity. The cortical networks for both PPI and IFM for the different expertise groups are shown in Fig. 8. Differences between the graph isolation approaches were not significant ($\Delta c{\mathrm{HbO}}_{2}$: two tailed t-test: t-difference: 3.508, df-t: 58.8; p=0.99; $\Delta c{\mathrm{HbO}}_{2}$: two tailed t-test: t-difference: 5.369, df-t: 89.5; p=1).

### 3.5 Manifold Analysis

Finally, we illustrate how IFM can be used to quantify group differences and exposed additional insight. The naive brain does not engage in the task exhibiting low response. Later, as the brain is encoding the task, neural activity and thus hemodynamics increases. Finally, upon learning, the brain becomes more efficient; the task-associated activity becomes more focused, and the hemodynamics after the execution of a learned skill is attenuated. This nonmonotonic hemodynamic response curve to surgical skill acquisition has been reported with fNIRS.^18^

**Fig. 9:**
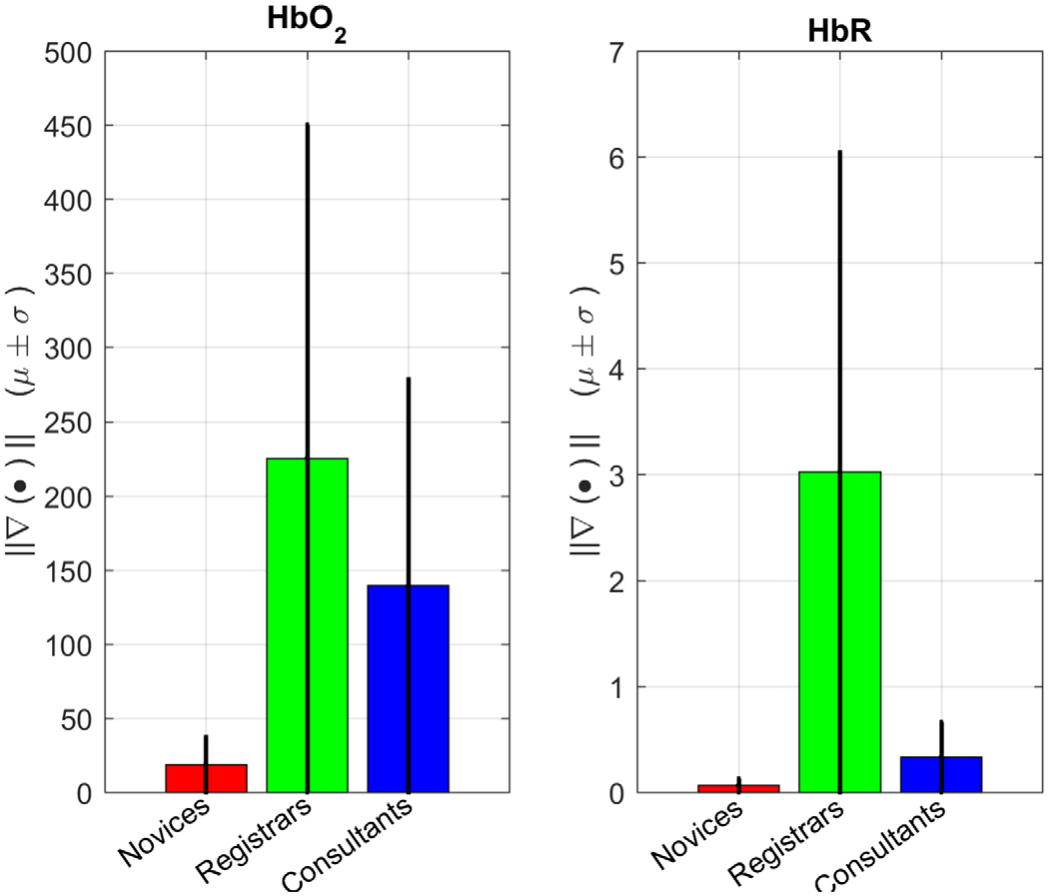
Barplots summarizing the norm of the gradient associated to expertise groups using the Marten RBF. Mean and standard deviation (error bars) are shown across replications and different trainings. We interpret higher gradients as the brain experiencing more abrupt changes.

When the points in the manifold are tagged by the experimental groups, the group loci offers a clue as to what is happening with cerebral hemodynamics at the cohort level. Figure 9 shows the norm of the gradient being nomologically consistent with the previous evidence.^18^ In agreement with previous theory, differences were significant for both Hb species (ANOVA: $\Delta c{\mathrm{HbO}}_{2}$: F(2,117)=3.07; p<0.05; ΔcHbR: F(2,117)=3.35; p<0.05).

### 3.6 Validation with Synthetic Data

A two groups synthetic dataset was generated for further validation. A total of 30 synthetic neuroimages were simulated. The synthetic neuroimages are divided into two arbitrary groups. Each group is assigned an adjacency matrix that represents the underpinning groupwise functional brain connectivity graph. The nodes represent the observed channels, and those assumed actived are linked into a full co-activity clique. A boxcar is generated to encode stimulation periods. The channel clean responses are generated from the convolution of the stimulus boxcar and the HRF (a double gamma was used to represent the HRF^19^). Only active channels were made to respond to these synthetic stimulus.

Then, the ideal signals were corrupted with experimental fNIRS noise coming from resting state recordings. The fNIRS background noise was collected from six subjects in resting state, the measurements were collected from consenting healthy adult volunteers within our laboratory. This data were collected during a previous project on neurorehabilitation of our group with the ethical protocol approved by the Instituto Nacional de Neurología y Neurocirugía from México City (ID: INNN 113-14). Only one session was recorded per subject. Data were collected from 28 channels using a NIRScout (NIRx) at wavelengths 760 and 850 nm. Optodes were placed over the occipital lobe and the posterior border of the frontal lobe aimed at covering the primary visual and motor cortices. These resting state fNIRS recordings include all sources of noise commonly encountered in an fNIRS neuroimage including physiological noise, optode movement artifacts, system drift, etc. This is regarded as an representative model of noise, and synthetic datasets including this type of noise are referred in fNIRS literature as semisynthetic. Random subsets of 10 channels are picked from a given resting-state neuroimage to provide the background error for a synthetic neuroimage. The noise is added channelwise to the ideal signal and several noise levels were considered in our tests: 0%, 30%, 60%, and 100%, respectively.

The synthetic stimulus train (Boxcar) included five stimulation periods of 15 s stimulus followedc by 20 s of rest and other 20 s recovery. The synthetic signals have a sampling frequency of 1 [Hz] and were simulated for 4.5 min, for a total of 275 samples where. A total of 55 (=20+15+20) samples per block were used for analysis.

**Fig. 10:**
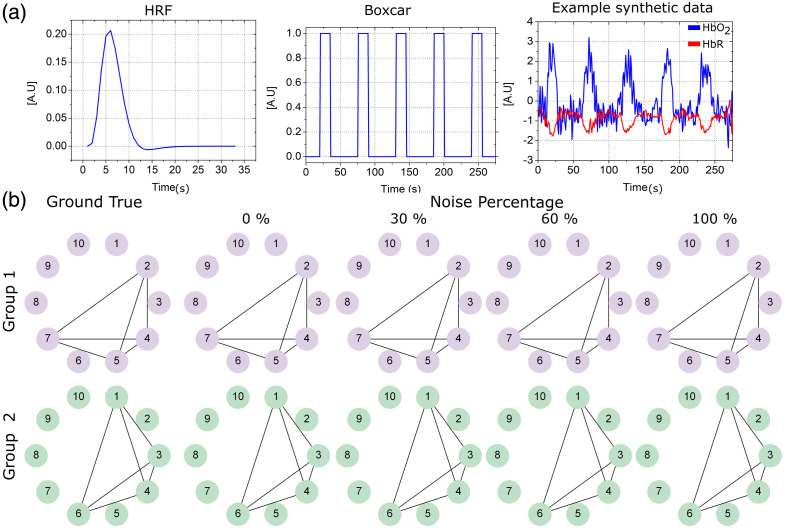
Validation on semisynthetic data. (a) The hemodynamic response function and the boxcar used, and an example of the semisynthetic data generated. (b) Validation on synthetic data. The ground truth networks for the two synthetic groups are shown on the left, and the recovered groupwise networks at different error levels are shown in the subsequent columns.

Figure 10 shows exemplary semisynthetic data, shows the ground truth graph per group, and illustrates the results. We depart from graphs with an arbitrary maximum size of 10 nodes with k active channels. Fifty replications were made. In each replication, a subset of k channels randomly selected between 2 and 7 were set to active (responsive to stimulus). Permutations of the potential graphs that are complete over the subset of k active channels were generated [Matlab combnk (10,k)], and a pair of them, one per group were chosen for the replication at hand. In each replication, a different underlying graph is chosen for each group. We further impose the condition for accepting a pair that they share no edges in common. The chosen graphs represent the groups for the replication. Once the pair of graphs have been generated, synthetic signals expressing the ground connectivity networks were forward generated. The ideal synthetic signals response (Boxcar* HRF) were contaminated with the experimental noise, by adding one resting-state recording, standardized and scaled (the selection of the resting state record is random). Depending on the number of channels being simulated every time, a subset of channels of the original resting-state records were randomly picked to be used as noise. Further, to incorporate additional variability, the resting-state signals were randomly phase shifted. Noise scaling followed a simple percentage over the canonical amplitude (0%, 30%, 60%, 100%). IFM succeded to recover the underlying connectivity network even with noise levels of 100%.

## 4 Discussion

We have presented IFM, a new tool for analysis of fNIRS neuroimaging. The research has provided the mathematical foundations of IFM and hints of alternatives when appropriate. In retrieving an analytical expression for the functional manifold, we opted for RBF and we have shown how four different RBF interpolants perform. Marten RBF interpolation offers a good compromise of error and smoothness. The interpolated surface exhibits a clear trend in both Hb species. With such a trend, it is easy to explore the ΔcHb surface by highlighting different subsets of interest from the cloud of points. Further, this trend strongly suggests that the $\Delta c{\mathrm{HbO}}_{2}$ and ΔcHbR surfaces lie on a differentiable manifold, an assumption shared by this and other previous related works.

The explicit analytic expression retrieved by IFM facilitated inference by allowing computation, rather than estimation by short hops, of groupwise gradients. Navigation over the manifold benefits from the new explicit model in principle permitting analytical derivation of the geodesic, instead of its approximation by short hops between topologically neighbor points.^8^ Other possibilities of IFM without altering its mathematical framework include but are not limited to single hemoglobin analysis, incorporation of additional physiological parameters, for example, the CCO, grouping channels for ROI-based analysis, separation of cerebral circuits, grouping complex experimental units (for example dyads for hyperscanning), separation of different experimental conditions, exploration of longitudinal dynamics, etc. All of these have been hinted throughout the paper.

Two graph isolation alternatives have been given to facilitate comparison with other popular connectivity analysis approaches in which the solution is encoded by a graph, instead of a manifold.

When the ground truth is known, IFM has been able to recover the exact networks. For the experimental dataset, the lack of an experimental ground truth means that the synthetic assumed truth used here (from the full set reconstruction) introduces a clear bias in our estimation of the error. Also, the trend found for this specific dataset may not necessarily be present in other datasets and we are not providing any theoretical guarantees for this to be the case. Several choices although not arbitrary admit discussion, for example, different projections to ambient space, different embeddings, and different interpolation approaches. Each choice comes with some assumptions that affect final interpretation. Validation against PPI only offers validity regarding functional connectivity analysis but the use of IFM for other types of research questions has not yet been validated.

Our correction for extracerebral physiological noise was based on a simple low pass filtering by decimation. While not ignored the issue of systemic influence, but this is far from optimal by nowadays standards, and a more aggressive treatment might have been more convenient. It is therefore very likely that scalp blood flow and other systemic physiological confounds could have influenced the retrieved connectivity networks. It is unclear if this effect will equally affect the compared modeling techniques. We believe a manifold-based analysis can bring benefits to complement the existing scalp surface topographical approaches based on classical regression models or graph analysis. This includes the use of a highly expressive mathematical object, the concomitant estimation of the complexity of the phenomenon by means of decoding its inherent dimensionality, the possibility of visualizing full dataset at one glance (by means of reduction of required), or the capacity to express different relations by means of manipulating the surface geometry (internal results not shown) among others.

### 4.1 Limitations

At this point, we have not conducted neither empirical tests nor theoretical work to explore limits of IFM with regards to the capacity to work with sparse vectors and low-rank matrices. Also, the minimum number of points needed is only known for flat surfaces, i.e., n+1 with n the inherent dimensionality of the surface. For more irregular surfaces, other considerations are needed which we have not explored. But this is perhaps not as critical as the clear price to pay is the error. Our testing indicates that even with 20% points (1284 points) the overall error was acceptable for an experimental dataset. Since one neuroimage brings many points to the manifold, this number in terms of neuroimages is not very demanding. The tests with the semisynthetic dataset were able to recover the exact network even in the presence of noise levels at 100% and with only 20 points (2 groups × 10 channels).

## 5 Conclusions

The expressivity of manifolds provides a complementary tool for understanding fNIRS-derived brain hemodynamics at the cohort level. We enriched a manifold-based representation of hemodynamic data with parametric surfaces to produce IFM. IFM can help in understanding cohort variations of brain hemodynamics derived from fNIRS neuroimages. The proposed tool uses ΔcHb locally for each observation and then exploits scalable RBF to construct an approximate solution to the assumed functional manifold. IFM is not bounded to segregational activity or integrational, e.g., connectivity, analysis, but here we focus on the latter only hinting the former. We further anticipate manifold-based neuroimaging analysis to be particularly helpful in hyperscanning scenarios, where classical approaches in which the experimental unit is a single subject may need specific adaptations.
